# The Treatment of Indolent Ulcer

**Published:** 1910-05-28

**Authors:** 


### Medicine.

### THE TREATMENT OF INDOLENT ULCER.

The use of powdered rhubarb root as an external application for indolent ulcer was recorded so long ago as 1793 by Everard Home, Assistant Surgeon to St. George's Hospital, and the value of the remedy has been recorded again recently by Sir Dyce Duckworth. Some years ago a case of complete paraplegia was admitted to St. Bartholomew's Hospital under his care, with one of the largest bed-sores he had ever seen. The sacral bones were laid bare, and the patient was much emaciated and in a very feeble condition. With a water-bed, good diet, and skilled nursing, gradual general improvement occurred, and signs of healing with more vigorous granulation began to appear in the sore. Charcoal poultices were employed at first; iodoform, various lotions, and resin ointment followed. Many weeks elapsed, and the improvement was still slow. The alleged virtues of rhubarb powder for indolent ulcers were then thought of, and this was used at each dressing by freely dusting over the surface of the sore from a pepper-pot. The improvement was rapid and very marked. Benefit was also derived from pressure brought to bear by strappings of adhesive plaster from side to side of the concave sore. In a few weeks there was so much amendment that the wound was nearly closed, and the patient was fit to be removed from the hospital.

Sir Dyce Duckworth mentions that he has heard of two other cases of indolent sores which have distinctly derived benefit from this treatment, though he supposes that modern surgeons and bacteriologists may regard such a practice as a source of possible contamination and sepsis. One cannot answer for the aseptic qualities of the ordinary pulvis rhei of the Pharmacopœia, but one can be satisfied with its healing properties in cases of indolent ulceration. There is no doubt that, if thought desirable, an aseptic preparation of it could be readily produced. The astringency of rhubarb is probably due to the presence of tannic and gallic acid in it. In cases of painful (irritable) ulcers, Sir Everard Home added a fourth or eighth part of powdered opium to the rhubarb. This treatment, in the case of extensive ulcers, is alleged to have produced purging. No such effect occurred in the instances described above.

